# Water–Aluminum Interaction as Driving Force of Linde Type A Aluminophosphate Hydration

**DOI:** 10.3390/nano13172387

**Published:** 2023-08-22

**Authors:** Anže Hubman, Janez Volavšek, Tomaž Urbič, Nataša Zabukovec Logar, Franci Merzel

**Affiliations:** 1 Faculty of Chemistry and Chemical Technology, University of Ljubljana, Večna Pot 113, 1000 Ljubljana, Slovenia; anze.hubman@ki.si (A.H.); tomaz.urbic@fkkt.uni-lj.si (T.U.); 2 Theory Department, National Institute of Chemistry, Hajdrihova 19, 1000 Ljubljana, Slovenia; 3 Department of Inorganic Chemistry and Technology, National Institute of Chemistry, Hajdrihova 19, 1000 Ljubljana, Slovenia; janez.volavsek@ki.si (J.V.); natasa.zabukovec@ki.si (N.Z.L.); 4 Faculty of Mathematics and Physics, University of Ljubljana, Jadranska 19, 1000 Ljubljana, Slovenia; 5 School of Science, University of Nova Gorica, Vipavska 13, 5000 Nova Gorica, Slovenia

**Keywords:** energy storage, aluminophosphates, hydration, adsorption, density functional theory, molecular dynamics

## Abstract

Linde type A (LTA) aluminophosphate is a promising candidate for an energy storage material used for low-temperature solar and waste-heat management. The mechanism of reversible water adsorption, which is the basis for potential industrial applications, is still not clear. In this paper, we provide mechanistic insight into various aspects of the hydration process using molecular modeling methods. Building on accurate DFT calculations and available experimental data, we first refine the existing empirical force-field used in subsequent classical molecular dynamics simulations that captures the relevant physics of the water binding process. We succeed in fully reproducing the experimentally determined X-ray structure factors and use them to estimate the number of water molecules present in the fully hydrated state of the material. Furthermore, we show that the translational and orientational mobility of the confined water is significantly reduced and resembles the dynamics of glassy systems.

## 1. Introduction

Due to alarming rates of global warming and increased energy requirements as a result of significant population growth, efficient energy storage and its production from renewable sources is of utmost importance [1,2]. Recent efforts to address these challenges suggest porous materials, e.g., zeolites or metal–organic frameworks (MOFs), as promising candidates for sorption-based energy storage [3,4,5]. An extensively studied class of microporous materials are aluminophosphates (AlPO_4_s), which have a plethora of polymorphs that can form one-, two- or three-dimensional pore frameworks [6,7]. One of the characteristics of AlPO_4_s is their ability to adsorb significant amounts of water. It has been suggested that this is due to the the highly hydrophilic character of aluminum sites of an otherwise charge-neutral framework, the formation of energetically favorable water clusters due to hydrogen bonding and the ability of the host framework to reversibly deform and accommodate guest water molecules [8,9,10]. The basic underlying principle that makes these characteristics relevant for energy storage is that as water is adsorbed the energy is released (unchargedstate), and, vice versa, as the material is dehydrated, the energy is stored for potential use (charged state) [11].

Water adsorption in AlPO_4_s has been extensively studied both experimentally and theoretically, see, e.g., [8,10,11,12,13,14,15,16,17,18,19]. Nonetheless it remains poorly understood on a molecular level. Complexity is especially severe for large-pore polymorphs where, for example, even a reliable determination of the number of water molecules present in the pores at different hydration levels becomes non-trivial. To understand the behavior of adsorbed water at the atomic-level resolution and its influence on the AlPO_4_ framework, computer simulations corroborated by experimental data play a pivotal role. Density functional theory (DFT) calculations, praised as being unbiased, are time consuming and often difficult to converge due to large vacuum regions inherently present in porous materials. Therefore, DFT calculations are mostly restricted to polymorphs with small unit cells [20,21], and, in the case of ab initio molecular dynamics (AIMD) simulations [22,23], to short trajectories, which raises concerns regarding the insufficient exploration of phase space and finite-size effects. As a consequence, classical simulations (MD or MC) based on empirical force-fields are usually employed, allowing the use of supercells and simulation lengths in the order of several nanoseconds [12,13,24,25,26,27]. If a classical force-field is to be used, it should be capable of describing host–guest interactions with sufficient accuracy and allow for the possibility of framework deformation to take place as a result of guest binding. For example, in the case of water adsorption it is well known that aluminum sites can bind up to two additional water molecules (see, e.g., [8,10]), a key fact that has often been ignored in previous simulation-based studies.

In this paper, we present a computational study of water adsorption in AlPO_4_-LTA based on structural data from solid-state NMR spectroscopy, synchrotron total scattering and water uptake measurements. It was experimentally demonstrated that AlPO_4_-LTA performs better in terms of water uptake and energy storage capacity than AlPO_4_-34, MOF-801 and MIL-160, which were previously regarded as the most promising candidates for sorption-based applications [11]. Both AlPO_4_-LTA and AlPO_4_-34 show similarly steep isotherms but with the important distinction that for the latter the mechanism of stepwise hydration along with crystal structures of all respective phases is fully known [8], while for the former the same process has remained elusive. In what follows, we use a combination of first-principles approaches and MD simulations to shed light on several aspects of the hydration process. Although we derive and validate the force-field parameters for AlPO_4_-34, whose structure is experimentally better known than that of AlPO_4_-LTA, we also use the same inter-atomic potentials for AlPO_4_-LTA, assuming sufficient structural similarities. We estimate the number of water molecules present in the fully hydrated structure of AlPO_4_-LTA and discuss the influence of confinement on the translational and rotational dynamics of water molecules.

## 2. Materials and Methods

### 2.1. First-Principles Calculations

First-principles calculations were performed at the DFT level of theory using the Vienna ab initio simulation package (VASP) [28,29,30]. A plane-wave basis set was used, setting the cutoff energy value to 450 eV. The interactions of the valence electrons with the ionic cores were taken into account by means of the projector-augmented-wave (PAW) method [31]. The electron exchange and correlation were described by the PBE generalized gradient approximation [32]. To correct for ill-described dispersion forces, the Grimme-D3 method [33] was used. Reciprocal space was sampled at the Γ-point, using the method of Monkhorst and Pack [34]. For electronic minimization, a robust combination of blocked Davidson and RMM-DIIS algorithms was used [35,36] and the convergence criterion was set to $10^{-5}$ eV, unless explicitly stated otherwise. Gaussian smearing was used in all calculations, adopting σ=0.01 eV. Reference structures of the dehydrated rhombohedral AlPO_4_-34 (6 Al, 6 P and 24 O atoms per unit cell) and its fully hydrated triclinic counterpart (6 Al, 6 P, 36 O and 24 H atoms per unit cell) were equal to the crystal structures as determined by Varlec and coworkers [8]. All first-principles calculations were performed on a single unit cell of AlPO_4_-34, since the size of the AlPO_4_-LTA unit cell would dictate a prohibitively large computational cost.

#### 2.1.1. Vibrational Density of States

The vibrational density of states (vDOS) was obtained using the finite-difference approach [37]. Reference structures were energy-minimized using the conjugate-gradient algorithm until the forces ($F_{\max}$) on all atoms dropped below $10^{-2}$ eV/Å^2^, followed by additional optimization with the quasi-Newton’s method, requesting $F_{\max}<10^{-6}$ eV/Å^2^. During the minimization procedure, the lattice parameters were kept fixed, allowing only atomic positions to vary. The optimized structure of the dehydrated AlPO_4_-34 was used to construct the Hessian matrix, by applying the central difference formula to approximate the derivative of the force with respect to the position. The magnitude of the ionic displacement was set to 0.015 Å and a tighter convergence criterion of $10^{-7}$ eV was used for electronic convergence. Normal mode frequencies ($\nu_{i}$) at the Γ-point and the corresponding eigenvectors follow from the diagonalization of the Hessian, and the vDOS, g(ν), is given as follows:

(1) $$g\left(\nu \right)=\frac{1}{(3N-3)\Delta \nu }\sum_{i=1}^{3N-3}\delta \left(\nu -\nu_{i}\right),$$

where *N* is the number of atoms.

#### 2.1.2. Atomic Point Charges

In order to estimate polarization effects due to the presence of water, electronic population analysis is needed. Bader charges as defined by the AIM theory [38] were computed for both dehydrated and fully hydrated AlPO_4_-34. Atomic positions and unit cell dimensions were kept equal to the experimental ones. Only the electronic minimization procedure was performed via VASP to obtain the electron density distributions, which were exported to and post-processed using the open-source code Bader, distributed by the Henkelman group [39,40,41,42]. The convergence of the Bader charges was checked by increasing the density of the Fourier mesh when computing the electronic density.

#### 2.1.3. Ab Initio Molecular Dynamics

AIMD simulations [28] were conducted for the fully hydrated AlPO_4_-34 at several different temperatures, ranging from T= 300 K to T= 600 K. Equations of motion were integrated according to the Verlet scheme using a time-step of 0.5 fs. Periodic boundary conditions were imposed in all directions. Starting from the reference experimental structures, the system was equilibrated for 50 ps at the target temperature in the canonical (NVT) ensemble using the Nose–Hoover thermostat [43,44]. Following the equilibration, the system was allowed to evolve for another 50 ps in the microcanonical (NVE) ensemble, and the trajectory was saved at each integration step for analysis.

### 2.2. Classical Molecular Dynamics Simulations

Classical all-atom MD simulations were performed using LAMMPS (version 23 June 2022) [45]. Equations of motion were integrated according to the velocity-Verlet scheme with a time-step of 0.5 fs, and the periodic boundary conditions were imposed in all directions. Interactions between the atoms constituting the porous framework were described by the BKS potential:

(2) $$U\left(r_{ij}\right)=A_{ij}e^{-Br_{ij}}-\frac{C_{ij}}{r_{ij}}+\frac{q_{i}q_{j}}{4\pi \epsilon_{0}r_{ij}},$$

with parameters $A_{ij}$, $B_{ij}$, $C_{ij}$ and partial charges $q_{i}$ kept the same as originally proposed [46]. Water was described by the explicit three-point TIP3P-Ew model [47], which is parameterized to be compatible with the Ewald summation method [37] that is used in this work to handle long-range interactions. Bond lengths (O-H) and angles (H-O-H) were held fixed using SHAKE [48]. Host–guest interactions were modeled as a sum of the Lennard–Jones (LJ) and electrostatic potential:

(3) $$U\left(r_{ij}\right)=4\epsilon_{ij}\left[{\left(\frac{\sigma_{ij}}{r_{ij}}\right)}^{12}-{\left(\frac{\sigma_{ij}}{r_{ij}}\right)}^{6}\right]+\frac{q_{i}q_{j}}{4\pi \epsilon_{0}r_{ij}}.$$

Using the LJ parameters as proposed by Koh et al. [49] for framework species and by applying the Lorentz–Berthelot mixing rules, we find no impact of hydration on the structure of the host. As will be shown in the following section, one has to properly account for the water coordination to certain aluminum sites in order to observe the associated framework deformation upon hydration as seen in experiments. We achieve this by introducing harmonic restraints,

(4) $$U=K{\left(r-r_{0}\right)}^{2},$$

between the chosen pairs of aluminum atoms and the oxygen atom of the respective water molecules. Subsequently, we also refine the LJ parameters to achieve the best match with respect to the experimental values of the lattice parameters and the AIMD-simulated radial distribution functions of the fully hydrated AlPO_4_-34. We note that a similar approach has been followed elsewhere [25,50]. The refined set of LJ parameters is listed in Table 1, whereas for harmonic restraints we use values K=4.0 eV/Å^2^ and $r_{0}=$ 1.65 Å.

In all simulations, initial structures were energy-minimized through 100 steps of the steepest descent method, followed by 10 ps of thermalization from T=10 K to the target temperature by rescaling the velocities, initially drawn from the Gaussian distribution. Constant temperature (pressure) was maintained using the Nose–Hoover thermostat [43,44] (barostat [51,52]).

#### 2.2.1. Initial Structure Preparation

For simulations of the dehydrated systems, the reference structures were taken from the experiments performed in previous studies [8,11]. In the case of the hydrated AlPO_4_-34, the crystal structure is also available [8]. Additionally, four water molecules anchored to the aluminum sites having octahedral coordination were identified and previously described restraints were introduced. The fully hydrated phase of the AlPO_4_-LTA is unknown. From the water uptake and ^27^Al solid-state NMR measurements, which were conducted following protocols described in [11], it was found that between 100 and 400 water molecules are present in the unit cell of the hydrated AlPO_4_-LTA and that approximately half of all aluminum sites assume an octahedral coordination. Consequently, the crystal structure of the dehydrated AlPO_4_-LTA was used as a basis and several initial structures were generated by randomly positioning a different number of water molecules, not discriminating between two types of cages. Additionally, half of all aluminum atoms meant to assume an octahedral coordination were randomly chosen and harmonic restraints were introduced between the respective Al site and the two closest water molecules.

#### 2.2.2. Force-Field Validation

We have tested whether the BKS force-field can satisfactorily reproduce the volumetric thermal expansion behavior of both dehydrated polymorphs. Several MD simulations were run in the temperature range between T=300 K and T=500 K. For AlPO_4_-34, a 3×3×3 supercell was used, while for AlPO_4_-LTA a single unit cell was sufficient. Following initial thermalization and subsequent equilibration in the NVT ensemble (≈50 ps), the systems were allowed to relax for 100 ps under NpT dynamics at the target temperature and pressure p=1 bar. During the isothermal–isobaric stage, time-averaged volumes ${\bar{V}}_{N}$ were determined from the second half of the production simulation. Additionally, we have also computed the vibrational density of states for both polymorphs at T=300 K by Fourier transforming the velocity autocorrelation function [37].

#### 2.2.3. X-ray Structure Factors

The comparison of the X-ray structure factors, as obtained from synchrotron experiments and their simulated counterparts, forms the basis to estimate the number of water molecules present in the fully hydrated state of AlPO_4_-LTA. As a starting point, short (≈0.5 ns) constant-pressure MD (T=300 K, p=1 bar) simulations of the hydrated 1×1×1 AlPO_4_-LTA were run, gradually increasing the number of water molecules from 160 to 320 and keeping the ratio of octahedral versus tetrahedral Al sites as stated. The results of this series (to be detailed later) indicate that no significant deviation from cubic symmetry occurs. Consequently, we set the lattice parameter to match the experiment (a= 23.7 Å) and performed all subsequent simulations in the NVT ensemble. To compute the structure factors S(q), three hydrated structures of 2×2×2 AlPO_4_-LTA were constructed, hosting 160, 240 or 320 water molecules per unit cell. Each system was simulated at T=300 K for 4 ns. Only the last 2 ns were considered for analysis. For reference, the dehydrated AlPO_4_-LTA was also simulated using the same protocol, but, this time, the lattice parameter was set to a= 23.95 Å. Experimentally measured structure factors were determined for powder samples; consequently, we use the orientationally averaged expression for S(q) [53]:

(5) $$S\left(q\right)=\frac{1}{\sum_{i=1}^{N}f_{i}^{2}}\sum_{i=1}^{N}\sum_{j=1}^{N}f_{i}f_{j}\frac{\sin(qr_{ij})}{qr_{ij}},$$

where *N* is the number of atoms and $f_{i}$ is the X-ray atomic form-factor approximated as follows:

(6) $$f\left(q\right)=\sum_{i=1}^{4}a_{i}\exp\left[-b_{i}{\left(\frac{q}{4\pi }\right)}^{2}\right]+c.$$

Constants $a_{i}$, $b_{i}$ and *c* were taken from International Tables for X-ray Crystallography [54].

Synchrotron data were collected at the Diamond Light Source on a dedicated X-ray Pair Distribution Function beamline (I15-1), using an X-ray wavelength of 0.161669 Åup to a maximum *q*-range of 25 Å${}^{-1}$. A hot air blower was used to collect the measurements between 300 and 423 K with a capillary tube opened on one end so that the water molecules could leave the system and the material gradually achieved a completely dry state.

#### 2.2.4. Translational and Orientational Diffusion

The influence of confinement on the dynamics of sorbed water was followed through changes in its translational and orientational mobility with respect to the bulk conditions. Restrained water molecules were not considered for this analysis, since, by construction, their motion is restricted to the vicinity of their anchoring site. Translational diffusion was followed by the computation of the mean-squared displacement, $\langle x^{2}\left(\tau \right)\rangle$, while for the orientational counterpart we computed the orientational correlation function, $C_{2}$, defined as follows [37]:

(7) $$C_{2}\left(\tau \right)=\langle P_{2}\left[e_{i}\left(\tau \right)\cdot e_{i}\left(0\right)\right]\rangle =\langle 3\cos^{2}\theta \left(\tau \right)-1\rangle ,$$

where $P_{2}$ is the second order Legendre polynomial and $e_{i}$ denotes a unit vector pointing along the OH bond, therefore specifying the orientation of the water molecule in the laboratory frame. In both cases, 〈…〉 denotes the time and the ensemble average over the 2 ns trajectory, which was previously used to compute the structure factors.

#### 2.2.5. Bulk Water as a Reference

Simulations of the bulk TIP3P-Ew water model were run at T=300 K in the NVT ensemble. Initially, 1500 water molecules were randomly placed in a cubic simulation box with L≈ 35.5 Å, corresponding to the density of approximately ρ=0.998 g/mL. An extensive equilibration period was followed by a 2 ns production run and the trajectory was saved every 50 fs for further analysis.

## 3. Results and Discussion

### 3.1. Force-Field Validation

**Figure 1:**
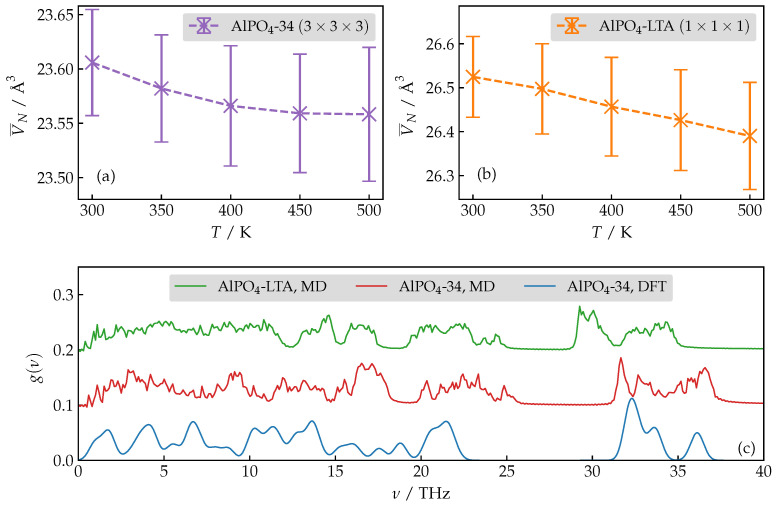
(**a**,**b**) Temperature dependence of the average volume of dehydrated AlPO_4_-34 and AlPO_4_-LTA, normalized with respect to the number of atoms. (**c**) A comparison of the vibrational density of states for both polymorphs as obtained by classical MD simulations at T=300 K and DFT calculations corresponding to T=0 K. Note that the vDOS is normalized as ∫g(ν)dν=1.

We begin our discussion by first analyzing the volumetric thermal expansion behavior of the dehydrated AlPO_4_-34 and AlPO_4_-LTA. It is well known that both polymorphs exhibit a *negative* thermal expansion coefficient ($\alpha_{V}$) [11,55]. Capturing such anomalous behavior is a strict test on the validity of the force-field. Here, we are interested in the sign of $\alpha_{V}$ and not in its absolute magnitude. In Figure 1a,b, we report the dependence of the time-averaged volumes with respect to the temperature. Clearly, the compression behavior is fully verified over a broad temperature range.

Additionally, we also demonstrate a comparison of the vibrational density of states for both polymorphs as obtained from force-field-based MD simulations and DFT calculations. We observe a satisfactory agreement between results computed using different levels of theory and different methodologies. Moreover, the similar features of AlPO_4_-LTA and AlPO_4_-34 vDOS serve as a justification of the transferability of the force-field.

### 3.2. Force-Field Optimization

It is well known experimentally that several of the water molecules adsorbed inside the AlPO_4_ pores reside in the vicinity of the hydrophilic aluminum sites at the distance of approximately 2.0 Å [8]. When such bonds are formed, the coordination polyhedron changes from a tetrahedron to a pentagonal bipyramid (one additional water molecule) or octahedron (two additional water molecules). This effect has often been ignored (with some notable exceptions) since the host was modeled as a rigid lattice or, according to our preliminary tests, existing force-field parameterizations of host–water interactions generate potentials to steep at too large distances to allow binding to take place. First, we focus on two questions: (a) Can the possibility of a water molecule exchange at the five- or six-fold coordinated Al site be neglected? (b) Should the charge polarization upon water binding be included in the description of interactions?

**Figure 2:**
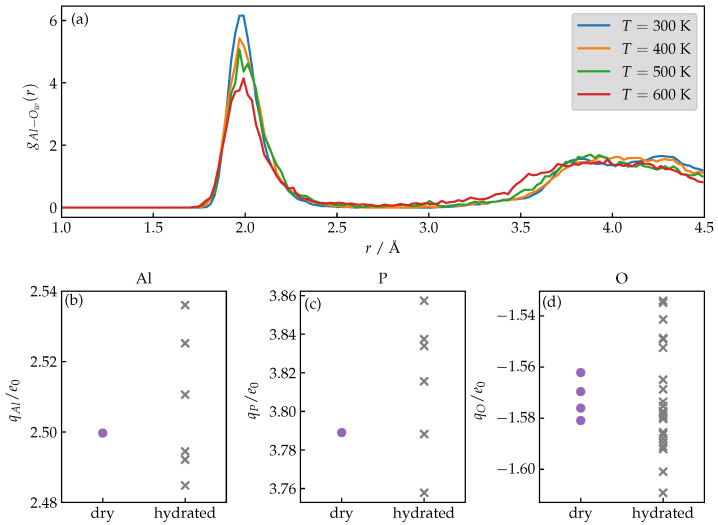
(**a**) Temperature dependence of the radial distribution function between the oxygen atoms of water molecules and Al sites for AlPO_4_-34 as obtained from AIMD simulations. (**b**–**d**) Atomic point charges according to Bader computed for Al, P and O, respectively, for framework atoms of hydrated and dehydrated AlPO_4_-34.

We seek the answer to the first question by analyzing the temperature dependence of the first peak of the radial distribution functions, $g_{Al-O_{w}}\left(r\right)$, between the oxygen atoms of water molecules and Al sites, computed via AIMD simulations. In Figure 2a, we observe that by increasing the temperature, which would in principle facilitate a possible water unbinding, a significant portion of H_2_Os in fact stays bound (a notable drop of the first peak is observed only above T=500 K). This observation indicates that one can neglect the ligand exchange at the specific Al site in the temperature range of 300–400 K and assume that, on average, water molecules stay permanently bound, justifying the use of harmonic restraints as stated in the Section 2.

To answer the second question, we compare the partial charges of framework atoms for hydrated and dehydrated phases of AlPO_4_-34, respectively. The presence of charge polarization would be reflected by a significant variation in the Bader charges as a result of charge reordering. Figure 2b–d unequivocally demonstrates that polarization is largely insignificant; computed values are spread rather than shifted and the largest changes are in the order of a few percent, indicating that static charges can be assumed and no additional description of polarization is needed.

Having laid the foundations, we now turn to the optimization of the force-field. Throughout the procedure, we keep all partial charges, the parameterization of the Buckingham potential and the TIP3P-Ew water model unchanged; the problem therefore reduces to the tuning of six LJ parameters plus the *K* and $r_{0}$. The latter two values are assumed to be the same for all relevant Al⋯OH_2_ contacts. The choice to tune only the framework–water interactions is based on preliminary tests, which show an exceptional sensitivity of other parameters to minor changes. As an initial guess for the LJ parameters, we adopt the parameter set by Koh et al. for framework species and apply the Lorentz–Berthelot mixing rules to obtain the host–guest parameters. Subsequently, we use a trial–error approach until a satisfactory agreement between the measured lattice parameters and the AIMD-generated pair distribution functions of the hydrated AlPO_4_-34 is achieved.

**Figure 3:**
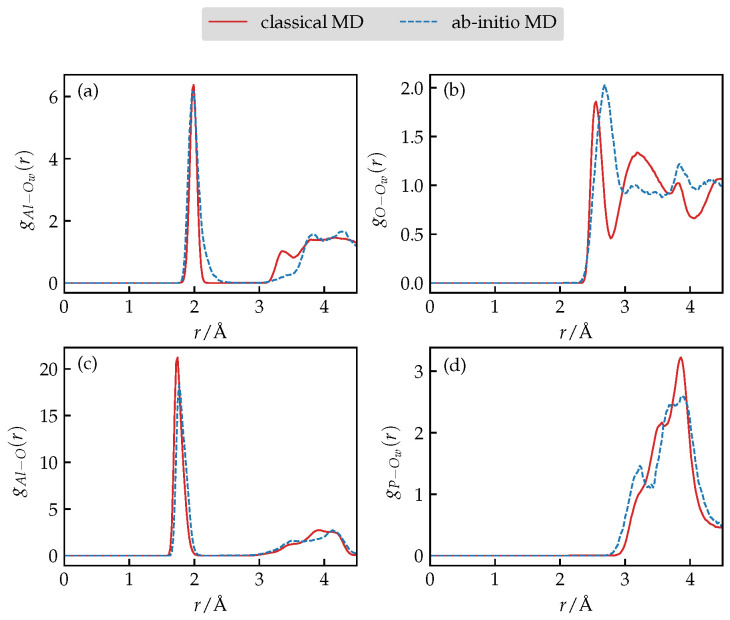
(**a**–**d**) A comparison of various partial pair distribution functions of the fully hydrated AlPO_4_-34 as obtained using AIMD or classical MD simulations after force-field refinement.

In Table 2, we demonstrate a comparison of the simulated lattice parameters of the hydrated AlPO_4_-34 using an optimized set of force-field parameters versus the experiment. A satisfactory agreement can be found with slight differences not exceeding a few percents. Most importantly, the shift from rhombohedral to triclinic symmetry, as observed in experiments, is fully captured. The same conclusion can be drawn from comparison of several partial pair distribution functions (Figure 3) where using refined parameters we manage to capture the main peaks as produced by the more accurate AIMD simulations. We add that during AIMD simulations the water molecule bound to a five-fold coordinated Al site was found to detach from said site and freely diffuse to the center of the pore. We suspect that this is either due to inaccuracies produced by the PBE exchange-correlation functional or due to large temperature fluctuations present in the system during the equilibration phase as a consequence of the Nose–Hoover thermostat and small simulation cell. Considering this fact, we did not restrain the specific water molecule during classical MD runs.

### 3.3. How Many Water Molecules Are Present in the Unit Cell of the Hydrated AlPO_4_-LTA?

**Figure 4:**
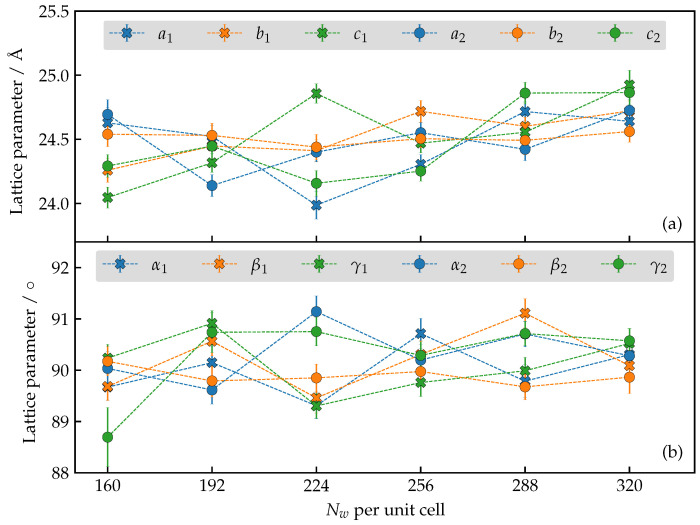
(**a**) Edge lengths of the simulation cell for various trial hydration levels. (**b**) Angles of the simulation cell for various trial hydration levels. In both cases, dotted lines are a guide to the eye.

It is not known experimentally whether the lattice parameters of AlPO_4_-LTA change significantly upon hydration. In Figure 4, we report the time-averaged lattice parameters for several hydration levels as obtained by constant-pressure MD runs. The span of the number of water molecules ($N_{w}$) assumed to reside in the unit cell of the host material is chosen to agree with the range estimated from water uptake measurements. For each hydration level, the results of two replicas of the simulated system are shown, differing in the initial configuration of the water molecules and the random choice of anchoring Al sites.

Despite the large range of plausible hydration levels, one observes that the major deformation concerning lattice parameters amounts to switching from a cubic to orthorombic crystallographic cell. Moreover, the random choice of anchoring sites does not seem to affect the general trends either. Both conclusions justify the simulation protocol that preserves the cubic unit cell, as stated in the previous section.

**Figure 5:**
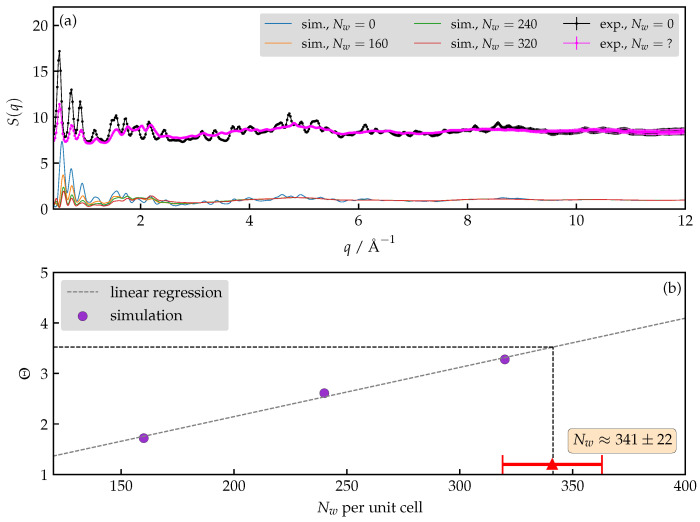
(**a**) The measured (upper) and the simulated (lower) X-ray structure factors of the hydrated and dehydrated AlPO_4_-LTA. (**b**) Linear correlation between the number of water molecules per unit cell of AlPO_4_-LTA versus the ratio (Θ) of the ∫S(q)dq.

Our next task is the estimation of the number of water molecules present in the unit cell of the fully hydrated AlPO_4_-LTA, which is performed via comparison of simulation-derived and experimental structure factors S(q). Computing the structure factors for several trial hydration levels (see Figure 5), we find an excellent agreement with the XRD measurements. The variation in the low-*q* region appears to depend on the number of the water molecules in the system. We can integrate S(q) in the selected region and define the ratio:

(8) $$\Theta =\frac{A_{dry}}{A_{hyd.}}=\frac{\int_{0.4}^{1.4}S^{dry}\left(q\right)dq}{\int_{0.4}^{1.4}S^{hyd.}\left(q\right)dq}.$$

We find a linear correlation between Θ and $N_{w}$ ($\Theta =kN_{w}+n$). Repeating the same calculation for the measured S(q), one obtains $\Theta^{exp}$. The estimate, $N_{w}^{exp}$, is calculated as follows:

(9) $$N_{w}^{exp}=\frac{1}{k}\Theta^{exp}-\frac{n}{k}=341\pm 22,$$

where the uncertainty is estimated using standard expressions for error propagation, neglecting the error in the fitting parameters *n* and *k* and the numerical integration. The only source of the uncertainty therefore stems from the experimental data; its exact value depends on the choice of the integration boundaries since one can notice that the error grows with an increasing *q*. Our selection of the integration interval was therefore tuned in order to minimize the uncertainty while still capturing the essential characteristics of S(q) in the low-*q* region. Interestingly, we find that the mean value of $N_{w}$ is rather insensitive with respect to the integration limits. Notice that the presented estimate agrees fully with the water uptake measurements.

### 3.4. Structure and Dynamics of Water under Confinement

**Figure 6:**
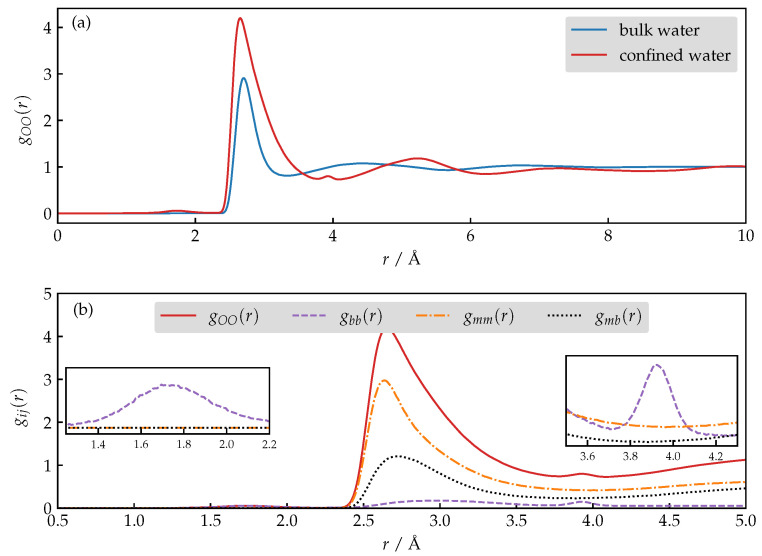
(**a**) Comparison of the $g_{OO}\left(r\right)$ of the bulk versus the confined water. (**b**) Decomposition of the $g_{OO}$ into contributions corresponding to the correlations between bound ($g_{bb}\left(r\right)$) and freely diffusing ($g_{mm}\left(r\right)$) water molecules, respectively, and the cross contribution ($g_{mb}\left(r\right)$).

In general, confinement perturbs structural and dynamical properties of the liquid compared to the bulk conditions. To probe the structural changes, we investigate the differences in the radial distribution function, $g_{OO}\left(r\right)$, of the adsorbed versus bulk water. Let us focus on Figure 6a where the $g_{OO}\left(r\right)$ of the adsorbed water corresponds to the situation where 341 water molecules are present per unit cell of AlPO_4_-LTA, thus obeying our mean estimate. We observe that, while both distributions show the same qualitative features, the ordering in the first and the second hydration shell is enhanced in the case of adsorbed compared to bulk water. Moreover, the second shell appears to be shifted to a larger *r* in the $g_{OO}\left(r\right)$ of the adsorbed case. Two additional weak signals appear in the $g_{OO}\left(r\right)$ of confined water, which require an explanation and confirmation that they are not an artifact produced by the force-field. Let us denote by $N_{m}$ the number of freely diffusing (mobile) and by $N_{b}$ the number of restrained (bound) water molecules; such a division is possible due to the construction of our simulation approach. One can decompose the $g_{OO}\left(r\right)$ into three contributions:

(10) $$g_{OO}\left(r\right)=\frac{N_{b}^{2}g_{bb}\left(r\right)}{{\left(N_{b}+N_{m}\right)}^{2}}+\frac{2N_{b}N_{m}g_{mb}\left(r\right)}{{\left(N_{b}+N_{m}\right)}^{2}}+\frac{N_{m}^{2}g_{mm}\left(r\right)}{{\left(N_{b}+N_{m}\right)}^{2}},$$

where $g_{bb}\left(r\right)$ and $g_{mm}\left(r\right)$ correspond to correlations between bound and mobile water molecules, respectively, while $g_{mb}\left(r\right)$ captures the correlations between both assigned types of molecules. The contributions are plotted in Figure 6b; one clearly sees that the discussed features originate strictly from $g_{bb}\left(r\right)$. The rationale that explains such behavior is that the first additional peak corresponds to two anchored water molecules when their dipole moments form a 90° angle, while the second additional feature describes a similar situation but this time the dipole moments form a 180° angle. Intermediate orientations, which are also present, are not obvious from the total $g_{OO}\left(r\right)$ since they contribute to the first and the most intense peak.

**Figure 7:**
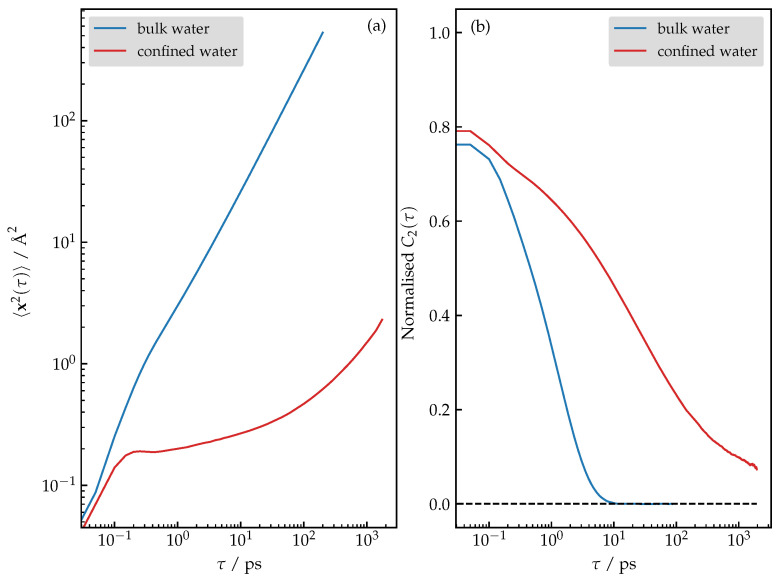
(**a**) Mean-squared displacement for bulk versus confined water. (**b**) Normalized orientational correlation function $C_{2}\left(\tau \right)$ for bulk versus confined water.

Lastly, we comment on the dynamical properties. While the mean-squared displacement (MSD) for the bulk water shows the expected normal diffusion, the confined water according to the shape of the $\langle x^{2}\left(\tau \right)\rangle$ in fact exhibits glass-like dynamics (see Figure 7a). Note that the behavior of the MSD for a larger τ is not the result of the poor sampling, which was verified by computing the MSD for two independent replicas of the system; the results are not shown for clarity. Additionally, analysis of the orientational correlation functions shown in Figure 7b reveals that the rotational mobility of the confined molecules is also significantly reduced.

## 4. Conclusions

In this work, we attempt to provide an interaction-based understanding of the complex process of water adsorption in AlPO_4_-LTA using MD simulations and DFT calculations. Through a thorough validation of simulation results against available experimental data, we identify the enhanced binding of water molecules to hydrophilic aluminum sites, leading to Al–O${}_{W}$ interatomic distances of about 2 Å, as a crucial component of the existing descriptions of the host–guest interactions responsible for the structural changes of the aluminophosphate framework upon hydration. While the existing force-fields have not been able to reproduce this effect, it is a contribution of the present work to consider the proper description with additional harmonic terms.

Based on refined force-field parameterization and comparison with experimental structure factors, we provide an estimate of the number of water molecules present in the structure of the fully hydrated AlPO_4_-LTA, which is 341 ± 22 molecules per unit cell. Moreover, the insight into translational and orientational diffusion properties of water molecules under confinement (except those bound to aluminum sites) reveals their glassy behavior. 

## Figures and Tables

**Table 1:** A set of refined Lennard–Jones parameters, applied in this work. Note that, by construction, hydrogen atoms of the TIP3P-Ew water model interact with the surroundings only via electrostatics.

Interacting Pair	$\epsilon_{\mathrm{ij}}$ / eV	$\sigma_{\mathrm{ij}}$ / Å
Al—$O_{w}$	0.0098423	3.798
P—$O_{w}$	0.0076489	3.242
O—$O_{w}$	0.0023926	3.453

**Table 2:** A comparison of the experimental versus simulated lattice parameters of 2×2×2 AlPO_4_-34 using an optimized set of force-field parameters. Relative error is given as $|\mu_{sim}-\mu_{exp}|/\mu_{exp}$, where $\mu_{i}$ denotes the mean value.

Lattice Parameter	Simulation	Experiment	Relative Error in %
*a* / Å	18.82 ± 0.07	18.052	4.2
*b* / Å	18.93 ± 0.05	18.676	1.4
*c* / Å	19.69 ± 0.07	19.016	3.5
α / ∘	96.3 ± 0.4	95.1	1.3
β / ∘	102.5 ± 0.5	104.1	1.5
γ / ∘	96.5 ± 0.4	96.6	0.1

## Data Availability

The data are available upon reasonable request.

## Abbreviations

The following abbreviations are used in this manuscript:

AIM	Atoms in molecules
BKS	Beest, Kramer, van Santen
DFT	Density functional theory
DOS	Density of states
LTA	Linde type A
LAMMPS	Large atomic/molecular massively parallel simulator
LJ	Lennard–Jones
MD	Molecular dynamics
MIL	Materials Institute Lavoisier
MOF	Metal–organic framework
NMR	Nuclear magnetic resonance
PAW	Projector augmented wave
PBE	Perdew, Burke, Erzernhof
TIP3P-Ew	Transferable interatomic potential, 3 point, Ewald
VASP	Vienna ab initio simulation package
XRD	X-ray diffraction
